# The use of an external ultrasound fixator (Probefix) on intensive care patients: a feasibility study

**DOI:** 10.1186/s13089-019-0140-9

**Published:** 2019-10-11

**Authors:** M. J. Blans, F. H. Bosch, J. G. van der Hoeven

**Affiliations:** 1 Department of Intensive Care, Rijnstate Hospital, PO box 9555, 6800 TA Arnhem, The Netherlands; 2 Department of Intensive Care, Radboud University Medical Center, PO box 9101, 6500 HB Nijmegen, The Netherlands

**Keywords:** Intensive care, Cardiac ultrasound, Cardiac output, Passive leg raising test, External

## Abstract

**Background:**

In critical care medicine, the use of transthoracic echo (TTE) is expanding. TTE can be used to measure dynamic parameters such as cardiac output (CO). An important asset of TTE is that it is a non-invasive technique. The Probefix is an external ultrasound holder strapped to the patient which makes it possible to measure CO using TTE in a fixed position possibly making the CO measurements more accurate compared to separate TTE CO measurements. The feasibility of the use of the Probefix to measure CO before and after a passive leg raising test (PLR) was studied. Intensive care patients were included after detection of hypovolemia using Flotrac. Endpoints were the possibility to use Probefix. Also CO measurements with and without the use of Probefix, before and after a PLR were compared to the CO measurements using Flotrac. Side effects in terms of skin alterations after the use of Probefix and patient’s comments on (dis)comfort were evaluated.

**Results:**

Ten patients were included; in eight patients, sufficient recordings with the use of Probefix could be obtained. Using Bland–Altman plots, no difference was found in accuracy of measurements of CO with or without the use of Probefix before and after a PLR compared to Flotrac generated CO. There were only mild and temporary skin effects of the use of Probefix.

**Conclusions:**

In this small feasibility study, the Probefix could be used in eight out of ten intensive care patients. The use of Probefix did not result in more or less accurate CO measurements compared to manually recorded TTE CO measurements. We suggest that larger studies on the use of Probefix in intensive care patients are needed.

## Background

The use of echocardiography by intensivists is rapidly growing [1]. Transthoracic echocardiography (TTE) is performed at the bedside, provides clinically relevant information and is safe in terms of radiation. TTE can be used to estimate dynamic parameters such as cardiac output (CO) but using TTE for these kinds of dynamic measurements is not without possible flaws [2].

To detect changes in CO using TTE, successive measurements of CO are needed. To get accurate results, the CO measurements should ideally be taken at exactly the same location.

**Fig. 1:**
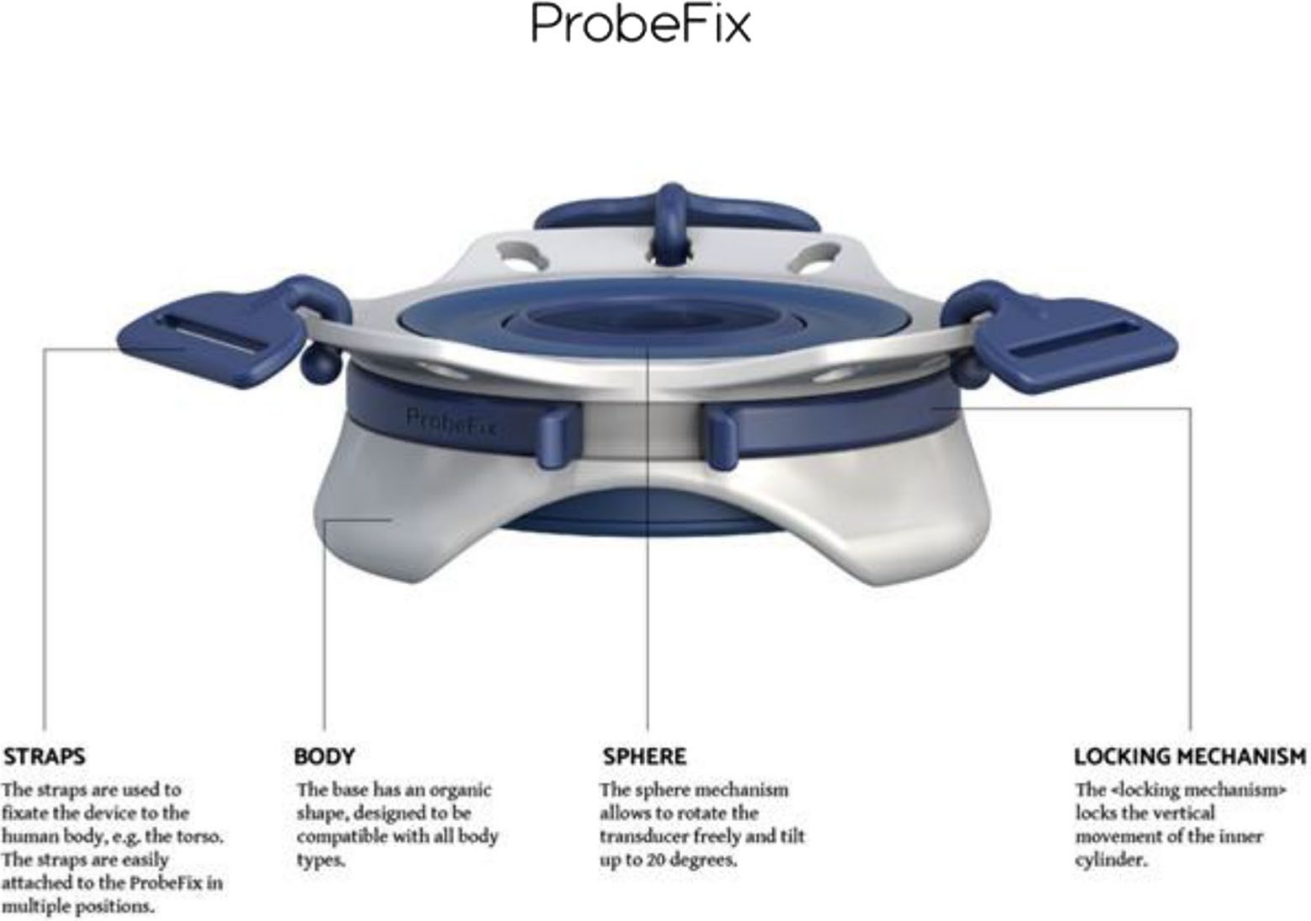
Description of the Probefix

**Fig. 2:**
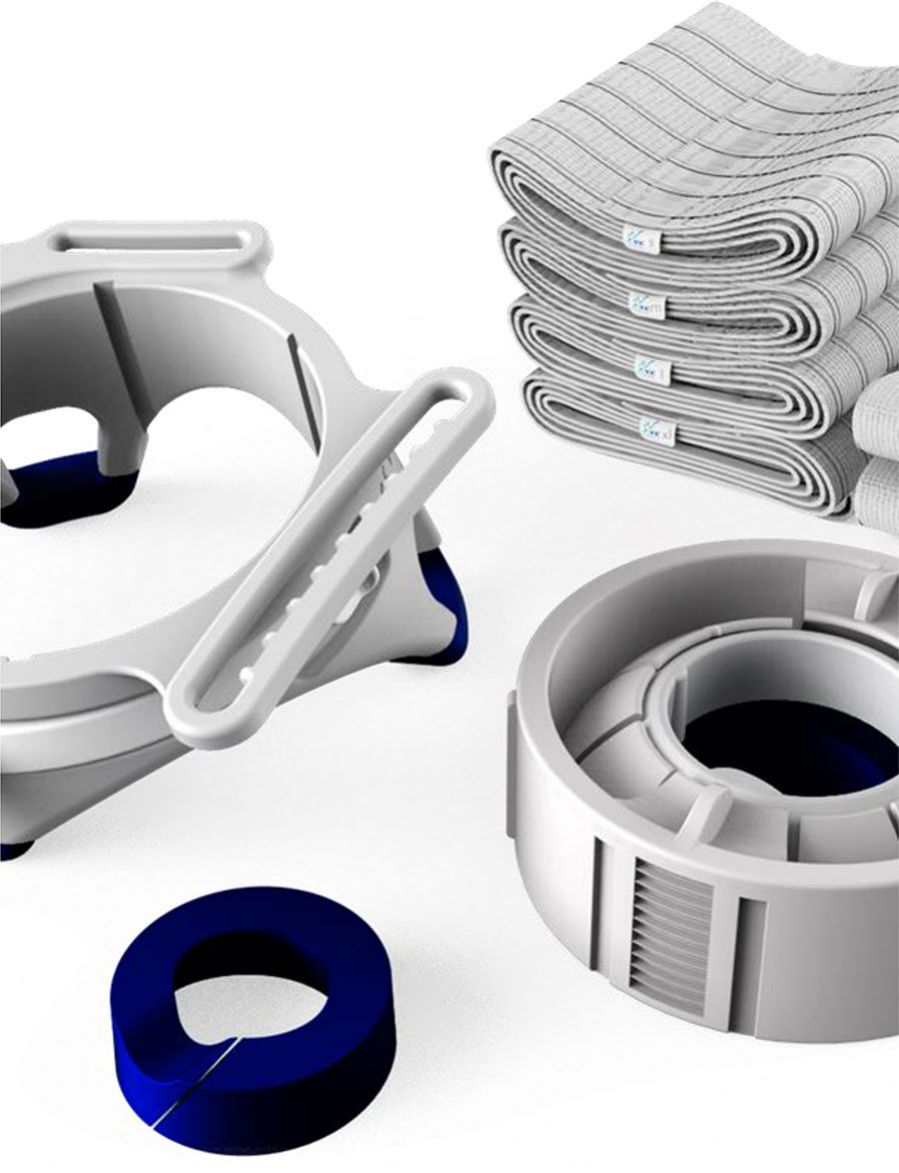
Components of the Probefix

**Fig. 3:**
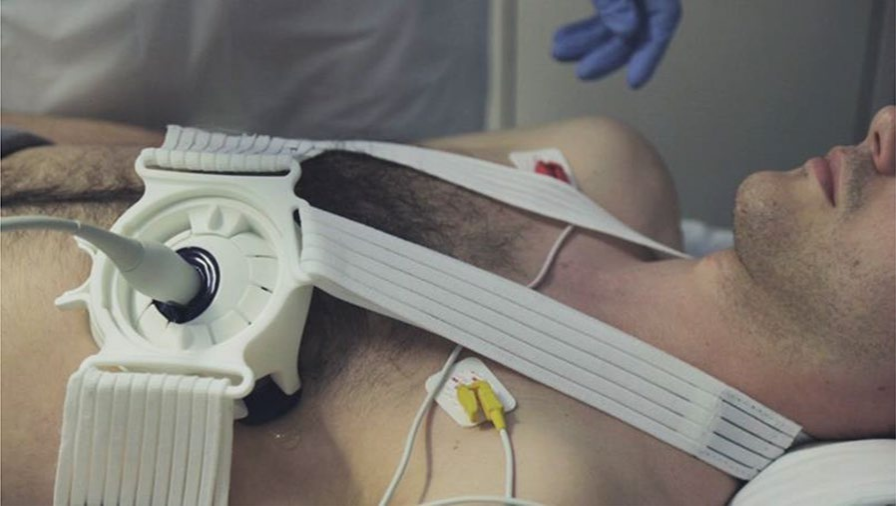
Example of the fixation of the Probefix on a patient

The Probefix is a non-invasive external ultrasound holder (Figs. 1, 2 and 3) which is strapped on to the patient with the use of non-traumatic straps.

When properly attached to the patient, the Probefix offers the opportunity to measure dynamic TTE parameters such as CO at exactly the same position thereby possibly increasing measurement accuracy.

To this date, there are no data on the use of the Probefix on adult intensive care patients. The Probefix has already been used in a study on adult outpatient cardiology patients [3] and another tailored holder for echocardiography was described in a study on 5 pediatric patients [4]. We designed a feasibility study to determine whether the Probefix can be used on adult intensive care patients for the purpose of measuring CO. CO with TTE with and without the Probefix was measured by using CO measurements made by the Flotrac as a reference. The Flotrac is a system that monitors CO by analyzing the systolic arterial pressure wave. The arterial catheter of the Flotrac system can be placed in the radial artery and has no need for pulmonary artery catheterization or other calibration [6].

Because there is variation between CO measurements between TTE and Flotrac, it will be difficult to assess the use of the Probefix during random CO measurements. It is more interesting to know whether possible changes in CO measurements will be the same after a therapeutic intervention such as a bolus of fluid.

**Fig. 4:**
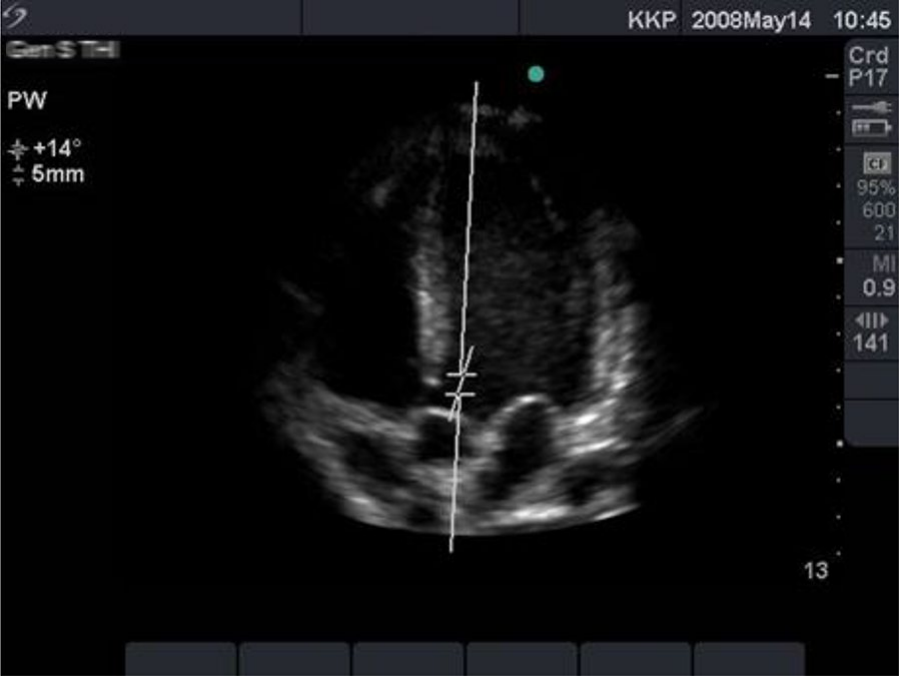
Apical 5 chamber view with pulsed wave Doppler sample volume

Besides CO, the Flotrac system monitors stroke volume and calculates the stroke volume variation (SVV). SVV is the percentage variation of stroke volume measured during a 20-s interval. Monitoring SVV can be helpful in detecting hypovolemia. When stroke volume variation is above 10%, hypovolemia is present [7]. When hypovolemia is detected with the Flotrac, a passive leg raising test (PLR) can be performed. During PLR, 150–200 mL of blood returns from the lower extremity veins to the central circulation [8]. With PLR, a reversible volume challenge is provided [9]. This is in contrast to an infusion of fluid (Fig. 4).

## Methods

In this prospective pilot feasibility study, the Flotrac system was used to detect possible hypovolemia (SVV > 10%) and Flotrac CO measurements were compared with the TTE CO measurements (with and without the use of the Probefix). CO was measured before and after a PLR test.

### Primary endpoints

Percentage of patients in which the Probefix can be used.

The correlation between the measurements done by Flotrac and TTE with and without Probefix.

Although no side effects are to be expected, we monitored the patients after the Probefix was removed for any skin damage. Possible skin damage was graded into three categories:

- No skin marks
- Mild skin marks (no treatment necessary)
- Severe skin marks (surgical or medical treatment necessary)

If awake, the patients were asked whether they felt the Probefix to be unpleasant on a scale of 0–10 (0 being: I did not feel anything and 10 being very unpleasant).

### Inclusion criteria

Adult intensive care patients (≥ 18 years), with hypovolemia detected with Flotrac (SVV > 10%).

### Exclusion criteria

Pregnancy, atrial fibrillation or other irregular heart rhythm, pulmonary edema, PLR not possible (e.g., neurological disease, spinal trauma, restricted limb movement, deep venous thrombosis, or any other reason as indicated by the attending intensivist) or age < 18 (years).

### Measurements

After detection of SVV of more than 10% by Flotrac, patients were eligible for inclusion and consent was asked. The TTE studies were done by 2 investigators (MB and FB) with Philips Affinity using the Phased Array probe; the patients were in supine position.

First in the parasternal long axis view (PLAX), the left ventricle outflow tract (LVOT) was measured in cm. Then, the following protocol was executed:

### Measurements with Probefix

*Step 1*: The Probefix is attached to the patient.

*Step 2*: An apical 5 chamber view was obtained with a 2.5-MHz probe. A pulsed wave Doppler sample volume was measured just below the aortic valve (Fig. 2), sweep speed: 150 mm/s.

A velocity time integral (VTI) was calculated by tracing the envelope velocity. CO measurements were calculated by combining this result with the measurement of the opening of the aortic diameter, obtained in the PLAX.

*Step 3*: PLR test.

*Stage 1*: raise the lower limbs of the patient to a 45° while the patient’s trunk is lowered in supine position.

**Fig. 5:**
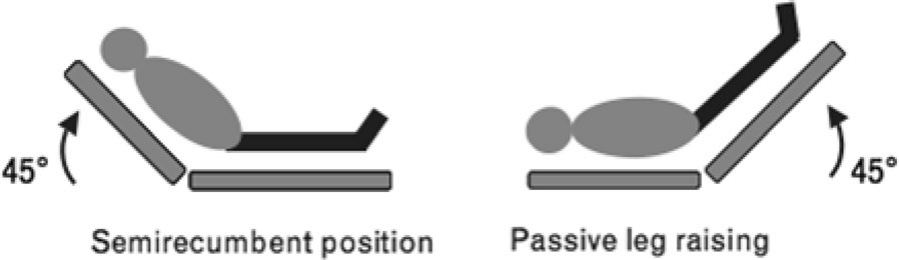
The two positions in PLR

*Stage 2*: after 1 min, measure the CO (Fig. 5).

*Step 4*: Steps 2–4 are repeated without the use of the Probefix.

### Measurements without Probefix

Repeat step 2 and 3.

### Statistical analysis

Primary study parameter(s)

1. Percentage of patients in which the Probefix can be used.
2. Scatter plots are constructed and the limits of agreement are calculated.

### Secondary study parameter(s)

Possible side effects of the Probefix.

Descriptive statistics are presented as mean with standard deviation for normally distributed continuous data.

The local ethic committee approved the study (NL62664.091.17); informed consent was obtained from all participants (or their next of kin) included in the study.

## Results

From May 2018 to April 2019, ten patients (six female and four male patients) were included.

In Table 1, patient characteristics are described.

**Table 1:** Patient characteristics

	Age	M/F	BMI	Diagnosis	Mode of ventilation	Probefix feasible?
Patient 1	70	M	23.1	Out of hospital cardiac arrest	Pressure Control	Yes
Patient 2	64	F	24	Out of hospital cardiac arrest	Pressure regulated volume control	Yes
Patient 3	69	F	23.4	Pneumonia	Pressure support	Yes
Patient 4	67	F	23	Chronic bronchitis	Pressure support	Yes
Patient 5	45	F	17.7	Pneumonia	Pressure support	Yes
Patient 6	59	M	19.4	Abdominal sepsis	Pressure support	Yes
Patient 7	68	F	24.2	Interstitial lung disease	Pressure support	Yes
Patient 8	61	M	24.5	Pneumonia	Pressure support	No
Patient 9	73	M	22.8	Pneumonia	Pressure control	Yes
Patient 10	63	F	25.4	Out of hospital cardiac arrest	Pressure support	No
Mean ± SD	63.9 ± 7.9		23.5 ± 3.1			

*M* male, *F* female, *BMI* body mass index, *SD* standard deviation

In eight patients (80%), the Probefix could be used properly leading to interpretable measurements. In two patients, insufficient views with the Probefix were obtained and in these patients, there were sufficient views using the TTE probe manually; so, the problem was Probefix related and not TTE related.

One patient was breathing spontaneously, three patients were on controlled mechanical ventilation and six patients received pressure support. Mean peak inspiratory pressure was 13 cm H_2_O (± 6.9), mean post expiratory end pressure 6 cm H_2_O (± 5.1), mean tidal volume was 480 mL (± 110) and mean LVOT was 2.1 cm (± 0.23).

**Fig. 6:**
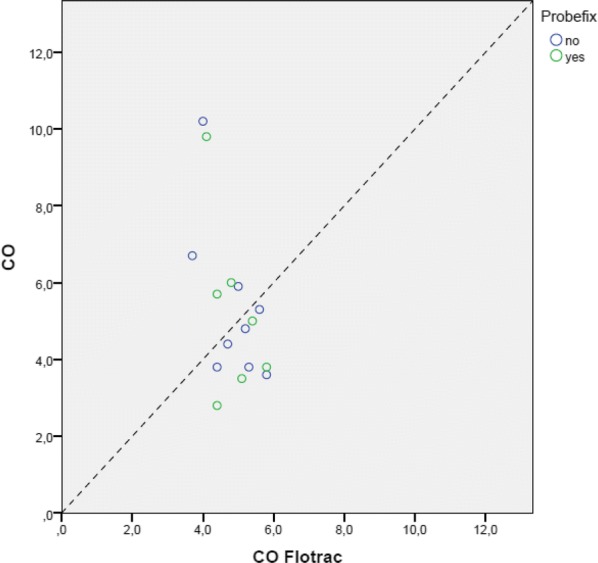
Scatter plot transthoracic echo (TTE) cardiac output (CO) vs. Flotrac with (green points) and without the use of Probefix (blue points)

**Fig. 7:**
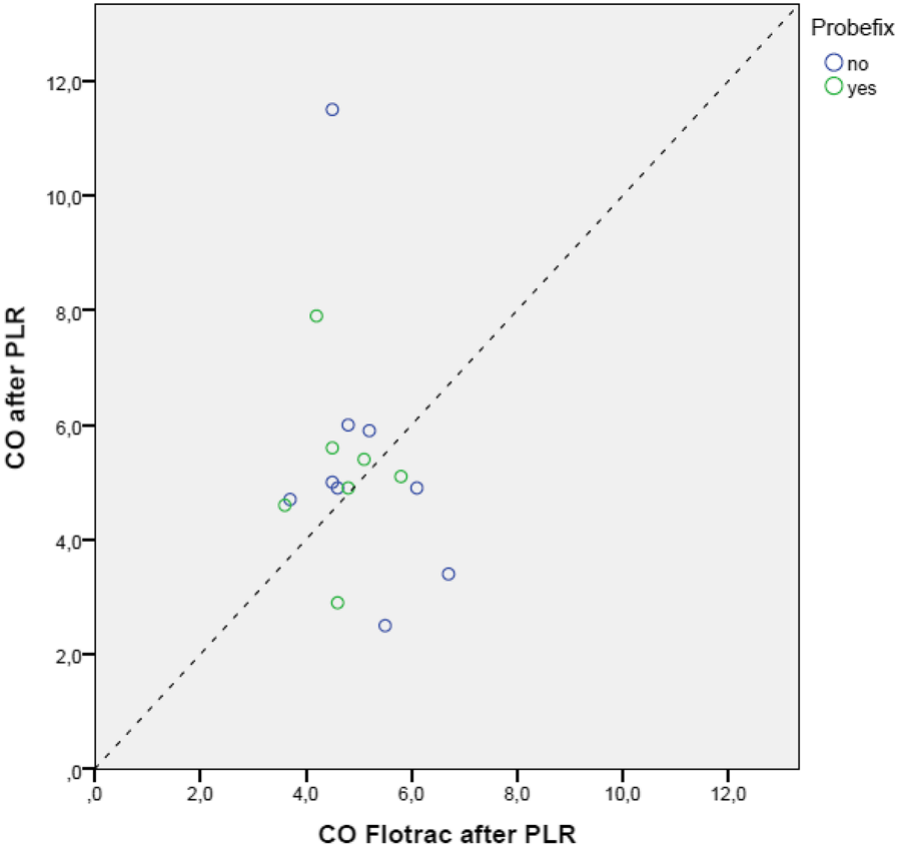
Scatter plot transthoracic echo (TTE) cardiac output (CO) vs. Flotrac with (green points) and without the use of Probefix (blue points) after passive leg raising (PLR)

When compared to the Flotrac measurements, there was no significant difference in correlation between TTE measurements with or without the use of the Probefix with the CO measurements done by Flotrac as a reference (Figs. 6, 7).

There were no serious skin lesions after the use of the Probefix. In four patients, there were minor skin marks that disappeared after some minutes. There was only one patient able to comment on the use of the Probefix and he reported only mild discomfort (2 on a scale of 10). In all patients, the time needed to process the protocol was within 15 min.

## Discussion

We demonstrated in this small feasibility study that the Probefix could be used in eight of ten adult intensive care patients. All but one patient (patient 6) were medical patients. Male and female patients were included and patients were both on controlled and support mechanical ventilation. When compared to CO measurements using Flotrac, there was no statistical difference between CO measurements by TTE with or without the Probefix.

Our study is small and as far as we know the first on adult intensive care patient in which the use of Probefix is described. As mentioned before, there is already a small case study on pediatric intensive care patients but none on adult intensive care patients [5.]

The number of included patients was too small to be able to conclude whether the use of the Probefix will lead to more accurate CO measurements but the opposite was also not found.

We know that measuring CO on intensive care patients using TTE is difficult in terms of comparison to other techniques such as thermodilution techniques [2] but it is feasible when done by intensivists [10]. In this study, only one heartbeat to measure CO using TTE was used which is fewer then normally performed but the aim of this study was primarily to assess the feasibility of the Probefix, not to establish the accuracy of the TTE CO measurements. By adding a PLR test to this study, we investigated also whether the Probefix could be used for dynamic measurements and using the PLR test, the possible side effects of volume loading were diminished.

In this study, TTE measurements were compared to the measurements done by Flotrac. We acknowledge the fact that there are doubts about the usefulness of pulse pressure analysis as a monitoring tool on the intensive care [11, 12] but we think that the Flotrac measurements could be used as controls to compare the TTE measurements with and without the use of the Probefix.

No skin injuries in the studied patients were found. Whether Probefix creates pressure sores with longer attachment periods needs to be investigated. Nine of ten patients could not comment on the (dis)comfort of the Probefix due to sedative medication so on this one comment only we cannot speculate on this aspect of the use of Probefix.

There are many interesting options for the use of Probefix; eventually, its use enables continuous non-invasive CO measurements.

For this development, we need software that can translate the LVOT-VTI TTE images to real-time CO. By optimizing TTE for continuous CO monitoring, the use of more invasive techniques can be greatly diminished making this development of interest for almost every patient in the intensive care unit.

## Conclusion

In this small feasibility study, it was shown that the Probefix can be used in eight out of ten adult intensive care patients for measuring CO also after a PLR test. More research is needed to further evaluate this new technique aiming at aspects such as accuracy, efficacy and costs.

## Data Availability

The datasets used and/or analyzed during the current study are available from the corresponding author on reasonable request.

## Abbreviations

TTE: transthoracic echo
CO: cardiac output
PLR: passive leg raising
SVV: stroke volume variation
PLAX: parasternal long axis
VTI: velocity time integral
LVOT: left ventricle outflow tract
